# The Relationship between Treatment Response and Overall Survival in Borderline, Non-Resectable and Resectable Pancreatic Cancer Patients Treated with Neoadjuvant FOLFIRINOX

**DOI:** 10.3390/jcm13175206

**Published:** 2024-09-02

**Authors:** Alex Barenboim, Diego Mercer, Kapil Sahnan, Alex Gaffan, Or Goren, Sharon Halperin, Eli Brazowski, Sharon Pelles Avraham, Joseph M. Klausner, Nir Lubezky

**Affiliations:** 1 Department of Surgery, Tel-Aviv Medical Center, Sackler School of Medicine, The Nicholas and Elizabeth Cathedra of Experimental Surgery, Tel Aviv University, Tel-Aviv 6997801, Israel; alex_barenboim@yahoo.com (A.B.); agaffan@gmail.com (A.G.); klausner.joseph@tlvmc.gov.il (J.M.K.); 2 Department of Radiology, Tel-Aviv Medical Center, Sackler School of Medicine, The Nicholas and Elizabeth Cathedra of Experimental Surgery, Tel Aviv University, Tel-Aviv 6997801, Israel; 3 Imperial College London, London SW7 2AZ, UK; kapil.sahnan@nhs.net; 4 Institute of Anesthesiology, Tel-Aviv Medical Center, Sackler School of Medicine, The Nicholas and Elizabeth Cathedra of Experimental Surgery, Tel Aviv University, Tel-Aviv 6997801, Israel; 5 Institute of Oncology, Sheba Medical Center, Ramat Gan 5262000, Israel; sharon.halperin1@gmail.com; 6 Institute of Pathology, Tel-Aviv Medical Center, Sackler School of Medicine, The Nicholas and Elizabeth Cathedra of Experimental Surgery, Tel Aviv University, Tel-Aviv 6997801, Israel; ebraz@tlvmc.gov.il; 7 Institute of Oncology, Tel-Aviv Medical Center, Sackler School of Medicine, The Nicholas and Elizabeth Cathedra of Experimental Surgery, Tel Aviv University, Tel-Aviv 6997801, Israel; sharonpa@tlvmc.gov.il; 8 Department of HPB and Transplant Surgery, Tel-Aviv Medical Center, Sackler School of Medicine, The Nicholas and Elizabeth Cathedra of Experimental Surgery, Tel Aviv University, Tel-Aviv 6997801, Israel

**Keywords:** pancreatic cancer, neoadjuvant, predictors of survival

## Abstract

**Background**: The National Comprehensive Cancer Network (NCCN)-recommended treatment for patients with borderline-resectable pancreatic cancer (BRPC) and locally advanced pancreatic cancer (LAPC) involves a combination of neoadjuvant FOLFIRINOX chemotherapy and the curative surgical resection of the tumor. This study seeks to identify the clinical, radiological, laboratory, and pathologic predictors that can anticipate the oncological outcomes of patients. **Methods**: In this study, we conducted a retrospective analysis of patients who had undergone curative surgical resection for BRPC, LAPC, or resectable disease with high-risk features after receiving neoadjuvant FOLFIRINOX at two institutions. We evaluated by means of multivariate analysis whether clinical and laboratory response, tumor markers, radiological response, and pathologic tumor response grade correlated with overall survival (OS) and disease-free survival (DFS). **Results**: The study enrolled a total of 70 patients with BRPC, LAPC, and resectable disease with high-risk features who underwent resection after neoadjuvant FOLFIRINOX. Age above 65 years and fewer than nine cycles of chemotherapy (OR 4.2; 95% CI 1.4–12.0; *p*-value 0.007); locally advanced tumors after neoadjuvant treatment (NAT) (OR 7.0; 95% CI 1.9–25.7; *p*-value 0.003); and lymph node disease and histological tumor regression grade 2 and 3 (OR 4.3; 95% CI 0.9–19.2; *p*-value 0.05) were risk factors linked to adverse OS and DFS. The median OS and DFS were 33 (22–43.9) months and 16.5 (11.3–21.6) months, respectively. **Conclusions**: Classification as a LA tumor after NAT was the only preoperative radiological factor that predicted adverse survival in patients undergoing curative surgery after NAT. Other clinical, biochemical, and radiological measures of response were not found to predict OS. Patient age, the cumulative administration of more than eight cycles of chemotherapy, and a significant pathological response were associated with better OS. The results of this study are important for treatment decision-making and prognostication in patients with BRPC and LAPC.

## 1. Introduction

Pancreatic ductal adenocarcinoma (PDAC) is the most common malignancy affecting the pancreas and oncological-including regional lymph nodes, and margin-negative surgical resection is the only therapeutic option that affords the opportunity for a cure [1,2]. There has been only a slight increase in survival rates in recent years. As a result, pancreatic cancer will become the second-leading cause of cancer-related deaths within the next decade [3].

The integration of a combined approach of complete surgical resection and adjuvant chemotherapy with modified FOLFIRINOX offers 3-year survival rates of 63.4% for patients with resectable tumors [4]. To optimize patient outcomes in those with borderline-resectable pancreatic cancer (BRPC), the National Comprehensive Cancer Network (NCCN) recommends initial neoadjuvant chemotherapy or chemoradiation [5]. Several studies have validated the safety of this treatment regimen in the preoperative phase [6,7,8].

The long-term cancer-related outcomes of borderline-resectable pancreatic cancer (BRPC) patients and select patients with locally advanced pancreatic cancer (LAPC) who receive neoadjuvant FOLFIRINOX treatment before undergoing curative resection have been found to be similar to those with resectable disease, according to several non-randomized studies [9,10,11,12,13].

Despite these promising findings, concerns remain regarding the potential for significant side effects and disease progression, resulting in the inability to achieve curative surgical resection in treated patients. The response to neoadjuvant chemotherapy is generally considered a positive prognostic marker for a cancer patient’s OS and disease-free survival (DFS) [14,15,16]. Treatment response can be assessed clinically, by means of tumor markers, radiologically, or histologically. However, definitions of response in each of these categories are not well-characterized for pancreatic cancer (PC), and the correlation with OS and DFS remains unclear [17,18]. In addition, recently published evidence highlights the significance of revised R status as an independent prognostic factor following NAT [19,20].

Our objective was to establish clinical, biochemical, radiologic, and pathologic responses to neoadjuvant chemotherapy and to assess the correlations of those definitions with OS and DFS among patients with BRPC and LAPC who underwent radical surgery after preoperative FOLFIRINOX.

By doing so, we hope to provide insights into how best to measure the efficacy of treatment by neoadjuvant chemotherapy in this patient population and to establish predictors of improved survival outcomes.

## 2. Methods

### 2.1. Patient Population

Ethical approval for this study was granted by the Institutional Review Board of the Tel-Aviv Medical Center and the Tel-Hashomer Medical Center. We collected and conducted a retrospective data analysis of prospectively collected data for all patients who received neoadjuvant FOLFIRINOX for resectable PDAC with high-risk features, borderline-resectable pancreatic cancer (BRPC) and locally advanced pancreatic cancer (LAPC) at both institutions between January 2013 and February 2020. Tumor resectability was defined according to NCCN criteria [5] (Supplementary Materials Table S1).

Inclusion criteria: Patients with borderline and locally advanced pancreatic cancer who had been treated by neoadjuvant FOLFIRINOX, and patients with resectable pancreatic cancer who had been treated by neoadjuvant FOLFIRINOX due to high-risk features (i.e., markedly elevated CA 19-9 levels, large primary tumor size, significant weight loss, severe pain and significant venous involvement thereby not fulfilling the criteria for BRPC).

Exclusion criteria: Patients who underwent upfront resection, those who started neoadjuvant FOLFIRINOX but did not complete at least three courses, and those who did not undergo any curative resection.

Primary outcomes: Overall survival and disease-free survival.

Secondary outcomes: Postoperative morbidity and mortality.

### 2.2. Initial Evaluation

Patients with pancreatic ductal adenocarcinoma (PADC) underwent triple-phase contrast-enhanced computerized tomography (CT). Resectability was determined by the pancreatic multidisciplinary team based on NCCN guidelines [5]. A specialist hepato-pancreatico-biliary radiologist reviewed all pre-treatment imaging studies to confirm the definition of tumors as BRPC or locally advanced pancreatic cancer (LAPC). The diagnosis was confirmed for all patients through biopsy before initiating NAT. As part of the initial evaluation, a chest CT and baseline measurements of CEA and CA 19.9 were performed.

### 2.3. Chemotherapy and Response Evaluation

Patients received FOLFIRINOX treatment as described [21], and the effectiveness of treatment was evaluated at regular intervals of 2–3 months using a combination of clinical assessments, CT imaging of the chest and abdomen, and measurement of tumor markers.

Biochemical response was specified as a drop in the tumor marker CA 19-9 after neoadjuvant FOLFIRINOX [17], and radiologic response was defined according to the Response Evaluation Criteria in Solid Tumors (RECIST) classification [22]. Treatment was continued for a median of 75 days (range 28–296) in patients who showed a positive response to therapy and had manageable side effects.

Surgery was conducted within six weeks following chemotherapy.

The criteria for local non-resectability were the presence of persistent long-segment arterial encasement following treatment and the assessment of non-feasibility for margin-negative resection both preoperatively by imaging and intraoperatively.

### 2.4. Surgery

All operations were performed by HPB surgeons specialized in pancreatic surgery. In order to determine the extent of the tumor, laparoscopic exploration was used to check for peritoneal and liver metastases, followed by an open exploration to assess arterial involvement. Operations performed on patients with borderline tumors after neoadjuvant treatment include the complete skeletonization of the SMA and the total resection of the mesopancreas. The ‘artery-first’ approach is used in our institution when there is suspicion of arterial involvement by the tumor and uncertainty about the feasibility of oncologic resection. If the tumor was adhered to the superior mesenteric vein-portal vein (SMV-PV), it was resected, and the vein was reconstructed. Short-segment involvement of the hepatic artery, celiac trunk, or superior mesenteric artery (SMA) also required resection and reconstruction. If the SMA was fibrotically encased, a biopsy was taken and sent for frozen section analysis. If cancer was found in the encasing tissue and the vessels could not be resected, the resection was aborted. However, if the frozen section analysis was negative for cancer, the vessel was skeletonized or resected.

### 2.5. Pathologic Characteristics

The histologic response grade to treatment was evaluated by an expert HPB histopathologist according to the protocol of the College of American Pathologists [23]. The CRM status is deemed positive when the tumor exhibits direct contact with or less than 1 mm from the radial margins [24].

Additionally, the assessment included tumor size, perineural invasion, lymphovascular invasion, surgical margin involvement, lymph node involvement and tumor regression grade (TRG). TRG categories range from TRG 0, which means complete pathological response, to TRG 3, which means no response to treatment.

### 2.6. Follow-Up

From the initiation of NAT, overall survival and disease-free survival were determined. An overall survival was defined as survival from the date of disease diagnosis to either the date of death or the last date of follow-up. Disease-free survival was specified as the time period from the operation to either disease recurrence or last follow-up in cases that remained disease-free.

Follow-up comprised abdominal and chest CT scans and tumor marker measurements conducted every 3–4 months during the first year, every 6 months in the second year, and annually thereafter.

### 2.7. Statistical Analysis

The original data were validated using descriptive tables. To explore potential relationships between dependent and independent variables, bivariate correlations were conducted using both Pearson and Spearman correlation coefficients. Pearson correlation was used for variables that were normally distributed, while Spearman correlation was applied to non-normally distributed or ordinal variables. In order to select variables for multivariate analysis, variables that showed a correlation with a *p*-value below 0.25 were selected for further analysis. This threshold was chosen to identify variables with a potential influence on the dependent variable, even if the relationship was not highly significant at this stage. Multivariate analysis was conducted using a backward step approach.

The correlation between different pre- and postoperative factors with overall and disease-free survival was assessed using the Kaplan–Meier (Log rank) test and Cox regression model. For all tests, a *p*-value less than 0.05 was deemed statistically significant. To address collinearity observed among certain parameters in the Cox regression model, we employed a strategy of combining correlated variables into a single parameter. ROC curves were utilized to calculate threshold values. The SPSS software package (version 23.0.0, SPSS Inc., Chicago, IL, USA, 2014) was used.

## 3. Results

**Figure 1:**
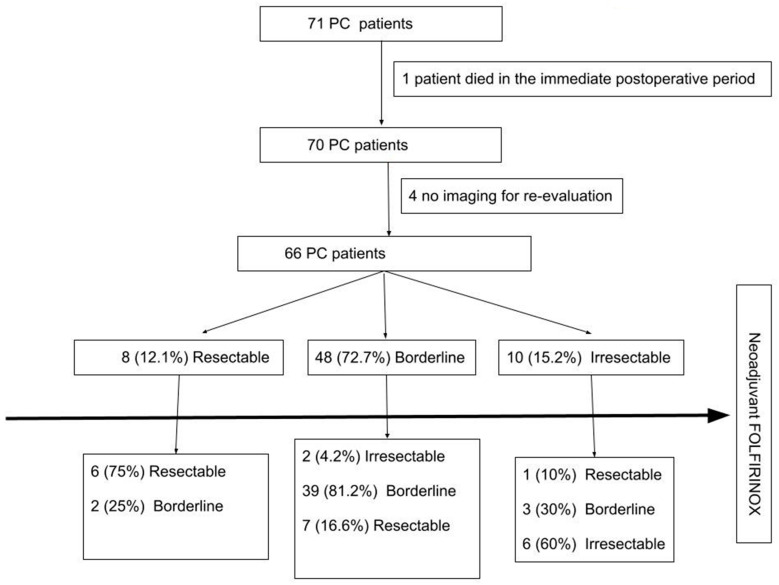
Flow chart of patients whose imaging studies depicted resectability after neoadjuvant FOLFIRINOX. PC—pancreatic cancer.

A total of 84 patients diagnosed with pancreatic cancer received treatment with neoadjuvant FOLFIRINOX followed by curative surgical resection between 2013 and 2020. Out of these, 47 of them were treated at the Tel-Aviv Medical Center and 24 others were treated at the Sheba Medical Center (Figure 1). Thirteen patients were found to have metastatic disease during surgery. One patient who had closed-loop small bowel obstruction was reoperated and died few days after in the Intensive Care Unit during the immediate postoperative period and was not included in the oncological analysis, leaving 70 patients for inclusion in the analysis. The median age of the cohort was 64 years (range 44–79). Table 1 lists the demographic characteristics of the patients at baseline. Tumors were located in the head of the pancreas in 47 out of 70 patients (67.1%). All study patients underwent endoscopic ultrasound-guided biopsies to confirm the diagnosis of pancreatic ductal adenocarcinoma, and those with jaundice underwent endoscopic retrograde cholangiopancreatography and stent placement. At the time of diagnosis, 48 out of 70 patients (68.6%) were classified as having borderline tumors, and 10 out of 70 (14.3%) were unresectable according to NCCN guidelines [5] (Supplementary Materials Table S1).

Eight out of seventy (11.4%) patients had resectable tumors with high-risk features. Those patients had significant involvement of the portal or mesenteric veins, which could compromise a R0 resection while not fulfilling the criteria for BRPC, and they were therefore referred for NAT.

### 3.1. Treatment

The study enrolled 70 patients with pancreatic cancer (PC) who received a median of six cycles of preoperative FOLFIRINOX chemotherapy. The median length of NAT was 75 days (28–296). Of these patients, 30% received at least eight courses before undergoing surgery, while 10% received neoadjuvant radiation. Adjuvant FOLFIRINOX was given to 42.8% of patients. The median total number of chemotherapy cycles was eight, and only 21.4% received 12 cycles, which is now considered the complete course of treatment. Most patients underwent pancreaticoduodenectomy (60%) followed by distal pancreatectomy (31.4%) and total pancreaticoduodenectomy (8.6%). All surgeries were performed via open surgery, and vein resection was performed in 45.7% of patients, while arterial resection was performed in 14.3% of patients.

### 3.2. Response to Treatment

#### 3.2.1. Radiologic Response

Radiological evaluation according to the RECIST classification (Table 2a) showed that 5.7% of patients had progressive disease, 40.0% had stable disease, 42.9% had a partial response, and 8.6% had a complete or near-complete response. The tumor diameter decreased in 72.8% of patients by a median of 25%, while 10.0% of patients had an unchanged tumor diameter (Table 2b). Anatomical downstaging (from LAPC to BRPC/resectable or from BRPC to resectable) was observed in 16.6% of all study patients (Table 2b). Detailed data for each case evaluation according to the NCCN guidelines are shown in Supplementary Materials Table S2.

#### 3.2.2. Clinical and Biochemical Response

Of the 70 patients, 47 (67.2%) presented with elevated levels of CA 19-9, with a median level of 105 U/mL at diagnosis. After NAT, the median CA 19-9 level was 35 U/mL, and 82.9% of patients with elevated levels of CA 19-9 had a decrease in their levels after treatment (Table 3). Out of seven patients with missing either pre- or post-treatment CA 19-9 values, two experienced a marker decline, as was cited in their medical records. Weight loss occurred in 53% of patients, with a median percentage of 5.83%. Albumin levels remained mostly unchanged, while lactate dehydrogenase levels increased at the end of chemotherapy (Supplementary Materials Table S1).

#### 3.2.3. Pathological Response

Of the 70 patients, 18.6% had a major pathological response defined as TRG 0 or 1. A negative R0 resection margin was achieved in 81.5% of patients (Table 4).

### 3.3. Postoperative Course

Nine patients (12.8%) had postoperative complications with a Clavien–Dindo level > 2, and 5.7% required reoperation due to severe sepsis or bleeding.

### 3.4. Survival

**Figure 2:**
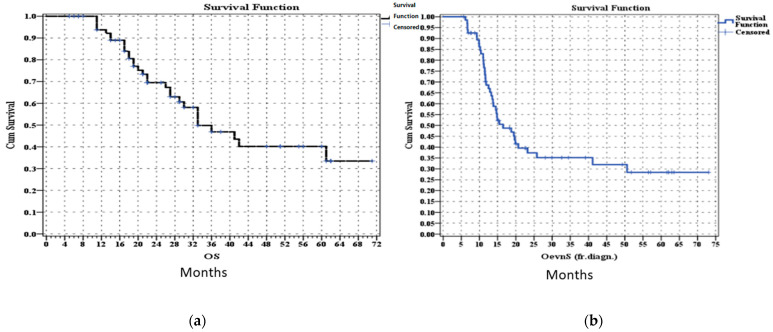
Kaplan–Meier curve of patients operated after neoadjuvant FOLFIRINOX. (**a**) Overall survival. (**b**) Disease-free survival.

The median overall survival (OS) was 33.0 (22–43.9) months, and the median disease-free survival (DFS) was 16.5 (11.3–21.6) months. Forty-one patients (58.6%) experienced recurrence after a median of 7.0 months, and at the last follow-up, 57.1% of patients were alive, with 70% of them being disease-free. The 1-, 2-, and 3-year OS rates were 94%, 70%, and 46%, respectively, as shown in Figure 2. None of the patients were lost to follow-up.

#### Predictors of Survival

An analysis of preoperative and postoperative factors affecting survival in patients with pancreatic cancer was conducted. The classification of the tumor as resectable/BRPC/LA before NAT was not found to be associated with prolonged survival. However, arterial involvement by tumor before NAT, LA status after NAT, patient age (cutoff 65 years), total number of chemotherapy cycles (threshold of eight or less), positive lymph nodes, and TRG 2 and 3 were associated with decreased survival in the multivariate analysis (Table 5a,b). Biochemical tumor marker response, including a normal CA 19-9 level before surgery or its normalization, did not provide a survival benefit.

Additionally, an assessment was made of radiologic response parameters with overall survival (OS). The analysis showed that LA tumors that were not down-staged predicted worse OS, while response according to RECIST and the additional anatomical parameters of radiologic response were not found to predict OS.

Our COX regression analysis revealed significant collinearity among several parameters, notably between positive lymph nodes and TRG 2 and 3, indicating a strong correlation with worsened survival outcomes. Similarly, advanced age (>65 years) in combination with fewer than nine chemotherapy cycles exhibited a similar trend towards diminished survival rates. COX regression models with combined parameters are presented in Table 5a,b.

**Figure 3:**
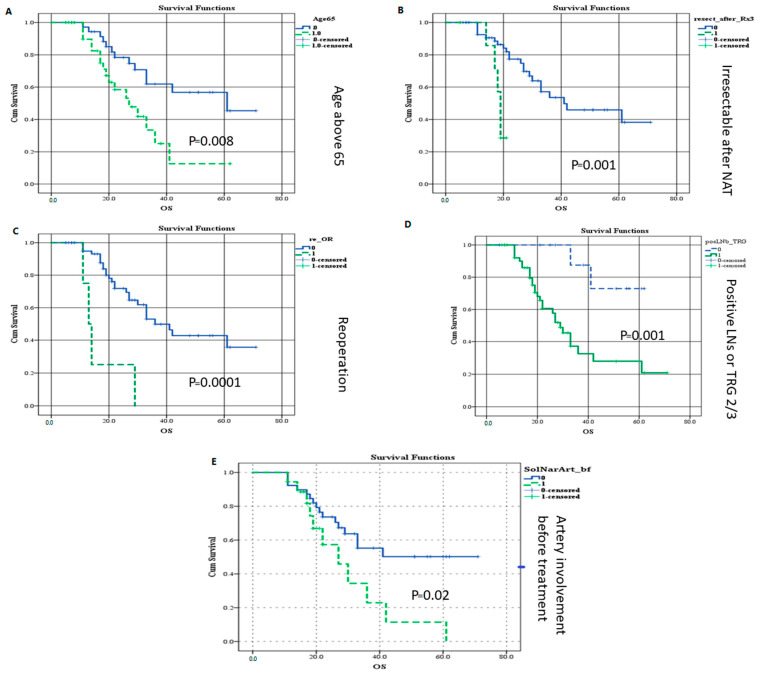
(**A**–**E**) Kaplan–Meier curves for cumulative proportion of patients with statistically significant factors influencing survival on multivariate analysis. (**A**) Age above 65 years. (**B**) Irresectable status after NAT. (**C**) Reoperations (**D**) Positive lymph nodes or TRG 2 or 3. (**E**) Arterial involvement by tumor before NAT.

Finally, a survival analysis was performed for all factors that appeared to be significant in the multivariate analysis, as shown in Kaplan–Meier plots (Figure 3).

We performed a multivariate analysis of survival factors pertaining to pancreatic tumors localized in the head, body, and tail of the pancreas, as detailed in Tables S3 and S4 in the Supplementary Materials. Many of the factors that demonstrated significance in that analysis maintained their significance, despite the challenges posed by relatively low statistical power.

## 4. Discussion

In the treatment of pancreatic ductal adenocarcinoma (PDAC), neoadjuvant therapy (NAT) is becoming more commonly employed to improve resectability and survival outcomes.

Recent randomized control trials have provided significant contributions to our understanding of pancreatic cancer treatment. Katz et al. [25]. The A021501 Phase 2 Randomized Clinical Trial showed that the addition of radiotherapy to neoadjuvant FOLFIRINOX did not demonstrate any advantage over neoadjuvant FOLFIRINOX alone. In PREOPANC-1 [13], 246 patients with either resectable or borderline-resectable tumors underwent upfront surgery followed by adjuvant gemcitabine or received neoadjuvant gemcitabine-based chemo-radio therapy followed by surgery and adjuvant gemcitabine. Although the initial results showed no significant difference in median OS (16.0 months with preoperative CRT versus 14.3 months with upfront surgery), longer follow-up revealed much better 5-year OS in the neoadjuvant group (20.5% vs. 6.5%) [26]. ESPAC-5 [27] showed that both regimens of short-course FOLFIRINOX or capecitabine-based regimens had better survival compared to upfront surgery.

A current clinical trial (Alliance [28]) is actively recruiting patients diagnosed with resectable pancreatic tumors to assess and compare the survival outcomes between neoadjuvant therapy (NAT) and upfront surgery followed by adjuvant treatment.

However, accurately predicting response to therapy, resectability, and prognosis remains a challenge in this patient population. Current studies, such as MESOPANC-1, intended to better define the mesopancreatic margin, are ongoing [29] This retrospective analysis of 70 PDAC patients who underwent resection after FOLFIRINOX NAT (including 7/70 [10%] who also had chemoradiation), with a mean follow-up of 27.8 months, found that age >65 years, eight or fewer cycles of perioperative chemotherapy, arterial involvement before NAT, positive lymph nodes at surgery, or failure to achieve a tumor regression grade (TRG) score of 0 or 1 were associated with a decreased overall survival (OS) rate. However, normal or normalized CA19-9 was not associated with survival benefits, and radiological parameters such as RECIST classification or the regression of venous involvement after NAT had no significant effect on survival. The only radiological parameters that were significant in the multivariate analysis were arterial involvement before treatment and classification as locally advanced pancreatic cancer (LAPC) after NAT.

These findings are consistent with previous studies that have also shown the limited ability of radiological response assessment to predict both resectability as well as prognosis in patients with PDAC receiving NAT. Ferrone et al. [30] did not find any prognostic significance of post-treatment imaging in 40 borderline and locally advanced patients. Katz et al. [31] did not find any association between RECIST response and median OS in 122 borderline-resectable PDAC patients. Similarly, Yasuta et al. [32] showed that the pathologic response of peripancreatic vascular invasion after NAT did not reflect the tumor response according to the RECIST classification of 29 borderline-resectable PDAC patients. These studies suggest that other factors beyond radiological response assessment may influence resectability and prognosis in PDAC patients receiving NAT.

The prognostic significance of the CA 19-9 response after NAT has been a subject of active investigation. The distribution of CA 19-9 values in our cohort aligns with the literature findings, with 20 patients (28.6%) exhibiting normal levels of the tumor marker at diagnosis, consistent with the data reported by Balaban et al. [33].

Our study did not find any prognostic significance of tumor marker response on survival. However, other studies have shown promising results. For example, in a study of 103 patients receiving FOLFIRINOX NAT, Heger et al. [17] identified a post-NAT CA 19-9 cutoff value at 91.8 U/mL, as well as a reduction to less than 40.7% of baseline, as independent predictors of resectability on multivariate analysis. A post-NAT CA 19-9 level < 91.8 U/mL was also found to significantly correlate with OS (34.3 vs. 18.3 months: *p* = 0.002) for the resected patients. Lee et al. [34] reported that patients who achieved a decline in CA 19-9 after NAT had significantly better DFS (10.9 vs. 6.8 months, *p* = 0.016), and a decline in CA 19-9 levels in a subgroup of patients with a baseline level of 37–1000 U/mL was associated with improved OS (HR: 0.262; 95% CI: 0.093–0.739, *p* = 0.011). Mellon et al. [35] found a significant correlation between tumor regression grade and the CA 19-9 value after treatment (*p* = 0.01) in 76 BRPC and 8 LAPC patients, but they observed no direct influence of CA 19-9 reduction or normalization on OS. Other studies have reported that a post-treatment decline in CA 19-9 of greater than 85% (Abbas et al. [36]) or a decrease in CA 19-9 levels by more than 30% (Veldhuisen et al. [37]) were associated with improved OS in resected patients after NAT.

Overall, these studies highlight the complexity of managing PDAC patients who receive NAT, as well as the need for personalized treatment approaches that consider the individual characteristics of the patient and the tumor.

Additionally, it should be noted that half of the group received adjuvant FOLFIRINOX, while the other half did not. Our intention is to administer a total of 12 cycles of FOLFIRINOX, although no established or proven protocol from randomized studies exists for providing adjuvant treatment to patients who have undergone neoadjuvant therapy followed by surgery [38].

While NAT can improve resectability and survival in some patients, it may not be effective in all cases, and additional research is needed to better understand the factors that influence response to therapy in PDAC. Further studies are also needed to evaluate the role of emerging biomarkers in predicting response to therapy and prognosis in this patient population.

Our study has several limitations that should be considered. Its retrospective nature precludes the possibility of completing missing data, it has a selection bias, and it is difficult to control confounding variables. The relatively small cohort size may reduce its statistical power. During the initial study period from 2013 to 2020, a significant proportion of patients in both centers was referred for upfront resection followed by adjuvant chemotherapy. Another limitation is that we do not have exact data on dose reduction during NAT.

However, as more scientific evidence became available, the number of patients treated with NAT followed by surgery increased. To address the potential bias introduced by this change in treatment approach over time, we performed Cox regression analysis. This statistical method takes into account the time distribution of events and not just the event values, helping to mitigate the impact of temporal variations in treatment strategies on our results.

There are some heterogeneities within the group because pancreatic head and pancreatic tail tumors have different oncological outcomes and require distinct surgical procedures.

Moreover, we did not include patients who underwent NAT and turned out to be metastatic during exploration.

Furthermore, the presence of missing data for some patients restricts certain analyses and may introduce bias in the results.

Additionally, it is important to note that the pathological evaluation of resection margins in our institution follows the guidelines of the AJCC 8th edition [39], which considers whether there is an involvement of inked resection margins or not. However, recent papers have proposed a classification of R0 as 1 mm from inked surgical margins being free of tumor, which may provide additional prognostic information [19,20].

Despite these limitations, our study still provides valuable insights into the studied population, and its findings can contribute to the existing knowledge in this field.

## 5. Conclusions

Our study demonstrates that neoadjuvant FOLFIRINOX treatment is a potential treatment option for patients with resected borderline-resectable pancreatic cancer (BRPC) or locally advanced pancreatic cancer (LAPC).

Additionally, patient age, the cumulative administration of more than eight cycles of chemotherapy, and a significant pathological response were associated with better overall survival. Notably, the only preoperative radiological factor found to predict adverse survival was classification as an LA tumor after NAT.

The results of our study contribute to the increasing amount of evidence advocating for the application of neoadjuvant FOLFIRINOX in the treatment of BRPC/LAPC and emphasize the significance of evaluating individual patient characteristics when determining the course of treatment.

## Figures and Tables

**Table 1:** Demographic characteristics of patients operated after neoadjuvant FOLFIRINOX.

Variable	Number
	(70 Patients)
Median age at diagnosis, years (range)	64 (44–79)
Males	45 (64.3%)
Tumor location	
Head/uncinate (right of SMV)	47 (67.1%)
Body/tail (left of SMV)	23 (32.9%)
Median neoadjuvant FOLFIRINOX cycles	6 (3–16)
Median length of NAT (days)	75 (28–296)
Adjuvant FOLFIRINOX	
Yes	30 (42.8%)
No	36 (51.5%)
Unknown	4 (5.7%)
Median total FOLFIRINOX courses (neoadjuvant + adjuvant)	8 (3–16)
Radiation before surgery	
Yes	7 (10%)
No	63 (90%)
Operation	
Radical pancreaticoduodenectomy	42 (60%)
Distal pancreatectomy	22 (31.4%)
Total pancreatectomy	6 (8.6%)
Venous resection	
Yes	32 (45.7%)
No	38 (54.3%)
Arterial resection	
Yes	10 (14.3%)
No	60 (85.7%)
Median length of stay (days)	10 (4–52)
Reoperation	4 (5.7%)
Postoperative complications with CD > 2	9 (12.8%)

CD—Clavien–Dindo, SMV—superior mesenteric vein, NAT—neoadjuvant treatment.

**Table 2:** Radiologic parameters of patients operated after neoadjuvant FOLFIRINOX.

(**a**)		
**Variable**	**Number** **(70 Patients)**	
Radiological downstaging by resectability		
Yes	11 (15.7%)	
No	52 (74.3%)	
Upstaging	3 (4.3%)	
Missing	4 (5.7%)	
RECIST classification		
CR + NCR	6 (8.6%)	
PR	30 (42.9%)	
SD	28 (40%)	
PD	4 (5.7%)	
Missing	2 (2.8%)	
Median percentage of diameter decrease in tumor	25 (0–100)	
(**b**)		
	**Before Treatment**	**After Treatment**
Resectability based on NCCN criteria		
Resectable	8 (11.4%)	14 (20%)
Borderline	48 (68.6%)	44 (62.9%)
Unresectable	10 (14.3%)	8 (11.4)
Missing	4 (5.7%)	4 (5.7%)
Median imaging diameter of tumor (mm)	35 (0–81)	25 (0–58)
Artery involvement at imaging		
Yes	24 (34.3%)	22 (31.4%)
No	33 (47.1%)	39 (55.7%)
Missing	13 (18.6)	9 (12.8%)
Vein involvement at imaging		
Yes	37 (52.8%)	24 (34.4%)
No	20 (28.6%)	37 (52.8%)
Missing	13 (18.6%)	9 (12.8%)

CR complete response, NCR near complete response, PR partial response, SD stable disease, PD progressive disease.

**Table 3:** Tumor markers of patients operated after neoadjuvant FOLFIRINOX.

Variable	Number
	(70 Patients)
CA 19-9 level before neoadjuvant FOLFIRINOX	
Normal	20 (28.6%)
Increased	47 (67.2%)
Missing	3 (4.2%)
CA 19-9 level after neoadjuvant FOLFIRINOX	
Normal	36 (51.4%)
Increased	30 (42.8%)
Missing	4 (5.8%)
Number of patients after chemotherapy whose CA 19-9:	
Decreased	39 (55.8%)
Increased	10 (14.3%)
Remained normal	16 (22.8%)
Missing	5 (7.1%)
Median CA 19-9 at disease onset (U/L)	105 (80–6989)
Median CA 19-9 after treatment (U/L)	35 (2–1981)
Median percentage of decrease of elevated CA 19-9 (U/L)	80.9 (1–99.6)

**Table 4:** Pathologic characteristics of patients operated after neoadjuvant FOLFIRINOX.

Variable	Number
	(70 Patients)
Type of tumor	
Adenocarcinoma	58 (82.9%)
Acinic carcinoma	1 (1.4%)
0 (complete response)	9 (12.9%)
Missing	2 (2.8%)
Median size of tumor (cm)	2 (0–4)
Median total lymph nodes	14.5 (1–21)
Positive lymph nodes	
Yes	25 (35.7%)
No	35 (64.3%)
Grade	
0	9 (12.8%)
Well-differentiated	10 (14.2%)
Moderately differentiated	31 (44.2%)
Poorly differentiated	15 (21.4%)
Missing	5 (7.4%)
Perineural invasion	
No	44 (62.8%)
Yes	22 (31.5%)
Missing	4 (5.7%)
Lymphovascular invasion	
Yes	13 (18.6%)
No	52 (74.3%)
Missing	5 (7.1%)
Radial margins involvement	
Yes	10 (14.3%)
No	57 (81.5%)
Missing	3 (4.2%)
TRG (CAP)	
0	9 (12.9%)
1	4 (5.7%)
2	30 (42.9%)
3	22 (31.4%)
Missing	5 (7.1%)

TRG (CAP)—tumor regression grade (College of American Pathologists).

**Table 5:** (**a**) COX regression analysis of factors influencing survival prediction of patients undergoing NAT and surgery for pancreatic cancer. Combined parameter is lymph nodes involved by tumor and tumor regression grade (TRG) 2 or 3. (**b**) COX regression analysis of factors influencing survival prediction of patients undergoing NAT and surgery for pancreatic cancer. Combined parameter is age above 65 years and number of chemotherapy cycles below nine.

(**a**)								
**Variable**	**B**	**SE**	**Wald**	**Df**	**Sig.**	**Exp(B)**	**95.0% CI for Exp(B)**	
							**Lower**	**Upper**
Age above 65 years	0.05	0.02	5.25	1	0.02	1.05	1.007	1.09
Nonresectable after NAT	1.98	0.64	9.43	1	0.002	7.2	2.04	25.4
Re-operation	2.06	0.70	8.59	1	0.003	7.8	1.9	31.07
Lymph nodes involved by tumor or TRG 2 and 3	1.47	0.76	3.71	1	0.05	4.3	0.9	19.2
Artery involvement at imaging before treatment	1.09	0.49	5.03	1	0.02	2.9	1.1	7.7
(**b**)								
**Variable**	**B**	**SE**	**Wald**	**Df**	**Sig.**	**Exp(B)**	**95.0% CI for Exp(B)**	
							**Lower**	**Upper**
Age above 65 years and number of chemotherapy cycles below nine	1.44	0.53	7.26	1	0.007	4.2	1.4	12.03
Nonresectable after NAT	1.95	0.66	8.72	1	0.003	7.04	1.9	25.7
Re-operation	1.42	0.72	3.92	1	0.047	4.1	1.01	16.8
Lymph nodes involved by tumor	1.92	0.49	15.42	1	0.0001	6.8	2.6	17.7
Artery involvement at imaging before treatment	1.09	0.49	5.03	1	0.02	2.9	1.1	7.7

## Data Availability

The datasets analyzed during the current study are not publicly available due to protecting patient privacy but are available from the corresponding author on reasonable request.

## Supplementary Materials

The following supporting information can be downloaded at: https://www.mdpi.com/article/10.3390/jcm13175206/s1, Table S1. Clinical and laboratory parameters of patients operated after neoadjuvant FOLFIRINOX; Table S2. Radiological parameters according to NCCN pancreatic cancer radiology reporting template; Table S3. COX regression analysis of factors influencing survival predictions of patients undergoing NAT and surgery for pancreatic cancer localized in the head of the pancreas. Combined parameter is age above 65 years and number of chemotherapy cycles below 9; Table S4. COX regression analysis of factors influencing survival predictions of patients undergoing NAT and surgery for pancreatic cancer localized in the body and tail of the pancreas. Combined parameter is age above 65 years and number of chemotherapy cycles below 9.
