# Supplementary material for: The Relationship between Treatment Response and Overall Survival in Borderline, Non-Resectable and Resectable Pancreatic Cancer Patients Treated with Neoadjuvant FOLFIRINOX
Source: J Clin Med. 2024 Sep 2;13(17):5206. doi: 10.3390/jcm13175206 (PMC11396552; doi:10.3390/jcm13175206)
Supplement: Supplementary file 1 [file jcm-13-05206-s001.zip › jcm-3154746-supplementary.pdf]

Supplementary Materials

- **NCCN resectability criteria:**

Resectable disease **- No arterial tumor contact (celiac axis [CA], superior mesenteric artery [SMA], or common hepatic artery [CHA]). No tumor contact with the superior mesenteric vein (SMV) or portal vein (PV) or ≤180° contact without vein contour irregularity.**
Borderline disease **- pancreatic head/uncinate process: Solid tumor contact with CHA without extension to the CA or the hepatic artery bifurcation allowing for safe and complete resection and reconstruction. Solid tumor contact with an SMA of ≤180°. Solid tumor contact with variant arterial anatomy (e.g.: an accessory right hepatic artery, a replaced right hepatic artery, a replaced CHA, and the origin of the replaced or accessory artery) and the presence and degree of tumor contact should be noted since any one of them may affect surgical planning. Pancreatic body/tail: solid tumor contact with a CA of ≤180°. Solid tumor contact with the SMV or PV of >180°, contact of ≤180° with contour irregularity of the vein or thrombosis of the vein but with suitable vessel length proximal and distal to the site of involvement allowing for safe and complete resection and vein reconstruction. Solid tumor contact with the inferior vena cava (IVC).**
Unresectable/locally advanced disease**: Head/uncinate process: Solid tumor contact >180° with the SMA or CA. Pancreatic body/tail: Solid tumor contact of >180° with the SMA or CA. Solid tumor contact with the CA and aortic involvement. Unreconstructible SMV/PV due to tumor involvement or occlusion (can be due to tumor or bland thrombus).**

Table S1. Clinical and laboratory parameters of patients operated after neoadjuvant FOLFIRINOX

**Variable**	**Number (70 patients)**
Median percentage of weight loss (%)	5.83 (0.04-20.2)
Weight during chemotherapy	
Gain	14 (20%)
Loss	37 (53%)
Stable	4 (5.7%)
Missing	15 (21.3%)
Median ALB at the beginning of the treatment, g/L	42 (27-48)
Median ALB at the end of the treatment, g/L	38 (28-46)
Median LDH at the beginning of the treatment, g/L	305 (155-731)
Median LDH at the end of the treatment, g/L	364 (149-938)

ALB - Albumin

Table S2. Radiological parameters according to NCCN pancreatic cancer radiology reporting template

**Parameter**	**Before treatment**	**After Treatment**

Appearance		
Hypoattenuating	49 (70%)	44 (62.8%)
Isoattenuating	6 (8.6%)	9 (12.9%)
Hyperattenuating	1 (1.4%)	0
Nonmeasurable	0	7 (10%)
Missing	14 (20%)	10 (14.3%)
Median diameter of tumor (mm)	35 (0-81)	25 (0-58)
Location		
Head/uncinate (right of SMV)	47 (67.1%)	
Body/tail (left of SMV)	23 (32.9%)	
Pancreatic duct narrowing/abrupt cutoff with or without upstream dilatation	42 (60%)	41 (58.6%)
Present	15 (21.4%)	20 (28.6%)
Absent	13 (18.6%)	9 (12.8%)
Biliary tree abrupt cutoff with or without upstream dilatation		
Present	21 (30%)	22 (31.5)
Absent	36 (51.4%)	39 (55.7%)
Missing	13 (18.6%)	9 (12.8%)
**SMA**		
SMA contact		
Present	26 (37.2%)	23 (32.9%)
Absent	31 (44.2%)	38 (54.3%)
Missing	13 (18.6%)	9 (12.8%)
Degree of solid soft-tissue contact		
≤180°	7 (10%)	9 (12.8%)
>180°	3 (4.3%)	3 (4.4%)
Absent	47 (67.1%)	49 (70%)
Missing	13 (18.6%)	9 (12.8%)
Degree of increased hazy attenuation/stranding contact		
≤180°	14 (20%)	11 (15.7%)
>180°	7 (10%)	4 (5.7%)
No	36 (51.4%)	51 (65.8%)
Missing	13 (18.6%)	9 (12.8%)
Focal vessel narrowing or contour irregularity		
Present	6 (8.6%)	1 (1.4%)
Absent	51 (72.8%)	60 (85.8%)
Missing	13 (18.6%)	9 (12.8%)
Extension to first SMA branch		
Present	2 (2.8%)	3 (4.4%)
Absent	55 (78.6%)	58 (82.8%)
Missing	13 (18.6%)	9 (12.8%)
**Celiac axis**		
Celiac axis contact		
Present	14 (20%)	13 (18.6%)
Absent	43 (61.4%)	48 (68.6%)
Missing	13 (18.6%)	9 (12.8%)
Degree of solid soft-tissue contact		
≤180°	4 (5.7%)	3 (4.4%)

>180°	2 (2.8%)	4 (5.7%)
No	51 (72.8%)	54 (77.1%)
Missing	13 (18.6%)	9 (12.8%)
Degree of increased hazy attenuation/stranding contact		
≤180°	5 (7.1%)	5 (7.1%)
>180°	6 (8.6%)	4 (5.7%)
No	46 (65.8%)	52 (74.4%)
Missing	13 (18.6%)	9 (12.8%)
Focal vessel narrowing or contour irregularity		
Present	2 (2.8%)	2 (2.8%)
Absent	55 (78.6%)	59 (84.4%)
Missing	13 (18.6%)	9 (12.8%)
**CHA**		
CHA contact		
Present	17 (24.3%)	14 (20%)
Absent	40 (57.1%)	47 (67.2%)
Missing	13 (18.6%)	9 (12.8%)
Degree of solid soft-tissue contact		
≤180°	7 (10%)	4 (5.7%)
>180°	4 (5.6%)	2 (2.8%)
No	46 (65.8%)	55 (78.6%)
Missing	13 (18.6%)	9 (12.8%)
Degree of increased hazy attenuation/stranding contact		
≤180°	7 (10%)	4 (5.7%)
>180°	3 (4.3%)	7 (10%)
No	47 (67.2%)	50 (71.4%)
Missing	13 (18.6%)	9 (12.8%)
Focal vessel narrowing or contour irregularity		
Present	5 (7.1%)	6 (8.6%)
Absent	52 (74.3%)	55 (78.6%)
Missing	13 (18.6%)	9 (12.8%)
Extension to celiac axis		
Present	1 (1.4%)	2 (2.8%)
Absent	56 (80%)	59 (84.4%)
Missing	13 (18.6%)	9 (12.8%)
Extension to bifurcation of right/left hepatic artery		
Yes	2 (2.8%)	2 (2.8%)
No	55 (78.6%)	59 (84.4%)
Missing	13 (18.6%)	9 (12.8%)
**Arterial variant**		
Variant anatomy		
Accessory right hepatic artery	2 (2.8%)	
Replaced right hepatic artery	9 (12.8%)	
Separate origin of hepatic artery	2 (2.8%)	
Replaced left hepatic artery	3 (4.3%)	
Arterial variant		
Present	16 (22.8%)	

Absent	41 (58.6%)	
Missing	13 (18.6%)	
Variant vessel contact		
Present	3 (4.3%)	4 (5.7%)
Absent	54 (77.1%)	57 (81.5%)
Missing	13 (18.6%)	9 (12.8%)
Degree of solid soft-tissue contact		
≤180°	0	1 (1.4%)
>180°	2 (2.8%)	2 (2.8%)
Absent	55 (78.6%)	58 (82.9%)
Missing	13 (18.6%)	9 (12.8%)
Degree of increased hazy attenuation/stranding contact		
≤180°	0	0
>180°	2 (2.8%)	3 (4.4%)
Absent	55 (78.6%)	58 (82.9%)
Missing	13 (18.6%)	9 (12.8%)
Focal vessel narrowing or contour irregularity		
Present	3 (4.3%)	2 (2.8%)
Absent	54 (77.1%)	59 (84.4%)
Missing	13 (18.6%)	9 (12.8%)
**MPV**		
MPV contact		
Present	25 (35.7%)	25 (35.7%)
Absent	32 (45.7%)	36 (51.4%)
Missing	13 (18.6%)	9 (12.8%)
Degree of solid soft-tissue contact		
≤180°	18 (25.7%)	17 (24.3%)
>180°	3 (4.3%)	0
No	36 (51.4%)	44 (62.9%)
Absent	13 (18.6%)	9 (12.8%)
Degree of increased hazy attenuation/stranding contact		
≤180°	10 (14.3%)	13 (18.6%)
>180°	5 (7.1%)	0
No	42 (60%)	48 (68.6%)
Absent	13 (18.6%)	9 (12.8%)
Focal vessel narrowing or contour irregularity (tethering or tear drop)		
Present	11 (15.7%)	6 (8.8%)
Absent	46 (65.8%)	55 (78.6%)
Missing	13 (18.6%)	9 (12.8%)
**SMV**		
SMV contact		
Present	37 (53%)	30 (42.9%)
Absent	20 (28.6%)	31 (44.2%)
Missing	13 (18.6%)	9 (12.8%)
Degree of solid soft-tissue contact		
≤180°	27 (38.6%)	20 (28.6%)
>180°	5 (7.1%)	3 (4.4%)

Absent<br>Missing	25 (35.7%)<br>13 (18.6%)	38 (54.3%)<br>9 (12.8%)
Degree of increased hazy attenuation/stranding contact		
≤180°	16 (22.8%)	17 (24.3%)
>180°	7 (10%)	3 (4.4%)
No	34 (48.6%)	41 (58.6%)
Missing	13 (18.6%)	9 (12.8%)
Focal vessel narrowing or contour irregularity (tethering or tear drop)		
Present	18 (25.7%)	11(15.7%)
Absent	39 (55.7%)	50 (71.4%)
Missing	13 (18.6%)	9 (12.8%)
Extension		
Present	5 (7.1%)	4 (5.7%)
Absent	52 (74.3%)	57 (81.5%)
Missing	13 (18.6%)	9 (12.8%)

SMA superior mesenteric artery, *CHA* common hepatic artery, *MPV* main portal vein, *SMV* superior mesenteric vein

Table S3. COX regression analysis of factors influencing survival predictions of patients undergoing NAT and surgery for pancreatic cancer localized in the head of the pancreas. Combined parameter is age above 65 years and number of chemotherapy cycles below 9.

Variable	B	SE	Wald	Df	Sig.	Exp(B)	95.0% CI for Exp(B)	
							Lower	Upper
Age above 65 years and number of chemotherapy cycles below 9	0.93	0.5	3.41	1	0.06	2.53	0.94	6.76
Nonresectable after NAT	2.22	0.76	8.64	1	0.003	9.19	2.09	40.36
Re-operation	1.87	0.7	7.07	1	0.008	6.46	1.63	25.57
Perineural invasion	1.77	0.65	7.49	1	0.006	5.86	1.65	20.79
Artery involvement at imaging before treatment	1.21	0.55	4.86	1	0.03	3.34	1.14	9.74

Table S4. COX regression analysis of factors influencing survival predictions of patients undergoing NAT and surgery for pancreatic cancer localized in the body and tail of the pancreas. Combined parameter is age above 65 years and number of chemotherapy cycles below 9.

Variable	B	SE	Wald	Df	Sig.	Exp(B)	95.0% CI for Exp(B)	
							Lower	Upper

Nonresectable after NAT	14.46	7.41	3.8	1	0.05	1.92	0.9	3.9
